# FloraMediX: AI-enabled recognition and therapeutic profiling analysis of indian medicinal flora

**DOI:** 10.3389/fbinf.2026.1864201

**Published:** 2026-07-07

**Authors:** R. L. Priya, Srushti Poriwade, Ananya Parthasarathy, Sri Haritha Movva, Isha Shinde, Archana Kumari Redhu, Pratik Chandrani, Vikram Gota

**Affiliations:** 1 Department of Computer Engineering Vivekanand Education Society’s Institute of Technology Mumbai, Mumbai, India; 2 Computational Biology & Crosstalk Lab, ACTREC, Tata Memorial Centre, Mumbai, India; 3 Department of Clinical Pharmacology, ACTREC, Tata Memorial Centre, Mumbai, India; 4 Training School Complex, Homi Bhabha National Institute, Mumbai, India

**Keywords:** artificial intelligence, deep learning, herbal medicine, image recognition, knowledge integration, medicinal plants

## Abstract

**Introduction:**

Accurate identification and profiling of medicinal plants are essential for integrating traditional herbal medicine into mainstream healthcare. However, plant identification remains challenging due to morphological similarities among species and the declining availability of taxonomic expertise.

**Methods:**

We developed FloraMediX, an AI-enabled platform that combines deep learning‐based visual recognition with structured pharmacological knowledge retrieval. The system employs a Swin Transformer Base (Swin-B) architecture for medicinal plant classification, a human-in-the-loop curation pipeline for iterative model refinement, and integration with phytochemical, therapeutic, and geographical databases including IMPPAT and OSADHI. The model was trained on 166,684 images representing 75 Indian medicinal plant species from the PlantNet-300K dataset and evaluated on an independent test set of 20,880 images.

**Results:**

The Swin-B model achieved a Top-1 accuracy of 93.70%, Top-5 accuracy of 99.68%, Macro F1-score of 91.38%, and an AUC of 0.9987. Performance exceeded baseline architectures, including ResNet-CBAM (90.57%), ConvNeXt (92.22%), and CoAtNet (92.22%) on the same evaluation dataset.

**Discussion:**

FloraMediX provides a reliable and accurate framework for medicinal plant recognition while simultaneously delivering curated phytochemical, therapeutic, and geographical information. The integration of advanced deep learning, expert-guided curation, and knowledge databases supports applications in research, education, biodiversity conservation, and the preservation of India’s traditional medicinal plant knowledge.

## 1 Introduction

Acknowledgment and use of medicinal plants are considered an essential component of India’s healthcare system. India is considered one of the 17 mega-biodiversity countries that contain more than 7,000 plant species known for their medicinal properties (National Medicinal Plants Board, 2026a), (National Medicinal Plants Board, 2026b). The domestic demand for medicinal raw plant drugs during the period of 2014–15 was estimated to be approximately 512,000 metric tons, while 195,000 metric tons were used in the herbal industry, and about 167,500 metric tons in the households in rural areas (National Medicinal Plants Board, 2026a). Medicinal plants continue to contribute significantly to modern pharmacology and drug discovery, with many therapeutic compounds originating from plant-derived sources (Dey et al., 2023). A national survey conducted by NSSO from July 2022 to June 2023 showed that almost 85% of households in rural areas and 86% of households in urban areas contained at least one person who recognized medicinal plants or folk medicine practice, whereas around 46% of rural individuals and 53% of urban individuals acknowledged using AYUSH (Ayurveda, Yoga and Naturopathy, Unani, Siddha, and Homeopathy) methods in the previous year (Press Information Bureau, 2023). These data show how much the population depends on herbal and traditional treatments, emphasizing the importance of efficient identification systems.

Identification and classification of medicinal plants are crucial in linking traditional knowledge to modern medicine. There is an abundance of medicinal plants in India, and thanks to the traditional medicinal systems such as Ayurvedic medicine, Siddha medicine, and Unani medicine, these plants have long been used for curative purposes. However, public awareness and knowledge regarding these medicinal plants have declined over time (Govindaprabhu and Sumathi, 2024). Identifying plants remains problematic due to similar morphology, intraspecies variation caused by environmental factors, and the loss of traditional knowledge. Misidentification may lead to ineffectiveness or harmful reactions; hence, there should be reliable classification systems available for identifying medicinal plants.

It should be noted that the recognition of traditional plant species poses challenges not only to the average person but also to the experts who work with plants on a regular basis. The traditional method of identifying plants includes the involvement of professional botanists and herbalists with many years of experience, gained in the process of education and practice. However, this approach also has some limitations because of its subjectiveness, time-consuming character (Khanh Le Dinh Viet et al., 2024), non-scalable nature, and inability to satisfy the increasing demand of modern society in terms of plant identification. In addition, uneven distribution of botanical information is crucial in remote and rural areas where medicinal plants are common. The identification of medicinal plants requires complex work on distinguishing between species of plants with identical morphological characteristics such as flowers, leaves, and stems (Tran Nguyen Nhut et al., 2024).

The development of deep learning technologies and computer vision tools has brought about revolutionary changes in automated recognition of plant species, offering unprecedented opportunities for democratizing botanical information and improving the accuracy of recognition. Studies have reported that computer usage, machine learning techniques, and deep learning technologies for the recognition and classification of medicinal plants provide the highest level of precision and reliability (Rahim et al., 2024).

Although CNN-based and hybrid deep learning techniques show a high degree of promise, several limitations need to be addressed before medicinal plant recognition systems can become useful in practice. First of all, most systems are oriented purely towards classifying species without using information about pharmacology, chemical makeup, and geography. Another problem faced by CNNs is that these models rely heavily on local feature extraction and cannot effectively capture long-range spatial dependencies or discriminate between morphologically similar plants in the presence of occlusions or changing environmental factors. Although ViTs have shown significant success in vision problems due to the ability of the attention model to capture the global structure of an image, these techniques are not sufficiently applied within the domain of medicinal plant informatics. Additionally, there is no effective human-in-the-loop approach to validate predictions and improve datasets by correcting inaccurate predictions. The proposed solution for overcoming these drawbacks is the creation of FloraMediX, which uses the Swin Transformer model architecture as an intelligent framework for medicinal plant identification and treatment profiling. The selection of the Swin Transformer architecture was mainly based on the use of hierarchical feature learning and shifted-window attention techniques to model both local and global structures effectively.

In this research, we address the aforementioned challenges by proposing an integrated intelligent platform which easily integrates visual recognition of the plant with extensive information on various types of medicinal herbs stored in the databases. The design of our platform involves using three types of integrated identification techniques: image scanning for field identification, name search for those with poor knowledge of plants, and regional searching by state to help identify local plants. With our proposed intelligent platform, we can bridge the identification-application gap in that we successfully link each recognized plant to relevant databases containing information regarding scientific nomenclature, taxonomy, geographical mapping, phytochemical constituents and medicinal uses from reliable sources, including Indian Medicinal Plants, Phytochemistry And Therapeutics (IMPPAT) (Database, 2025) database, Online Structural and Analytics based Database for Herbs of India (OSADHI) (CSIR–North East Institute of Science and Technology, 026) and Indian Pharmacopoeia.

The following is the outline for this paper: Part II presents a comprehensive literature review on current research trends related to deep learning-based plant identification tools, medicinal plant databases, and the issues that come with them. Part III presents the proposed architecture of the system, including methods for gathering data, preprocessing processes, creating the deep learning models, integrating the database, and designing the user interface. Part IV presents the experiment settings, evaluation metrics, and comparative performance analysis. Part V provides information about outcomes, implications, and future directions for optimizing system functionalities through multi-organ recognition, phytochemical prediction models, and integration with electronic medical records.

## 2 Related works

The area of automatic identification of medicinal herbs has witnessed substantial research in the past decade, which is due to advancement in deep learning models, availability of computing power, and realization of the critical need for readily available botanical information. In this chapter, we present a review of the latest academic work done in this area, analyzing their techniques, success and limitations in order to fit our system within this context (Wäldchen and Mäder, 2017).

The recent advancement of technology has resulted in the attainment of very high accuracy in the CNN-based medicinal plants recognition system. Bouakkaz et al. (2025), Widneh and Alemu, (2025) made use of Binary Chimp Optimization with residual blocks and Depth-wise Separable CNNs to develop optimal architecture models that attained over 99% accuracy with automatic feature extraction. With a total of 5,878 images, Mulugeta et al. (2024) evaluated seven architectures such as VGG16, VGG19, DenseNet201, ResNet50V2, Xception, InceptionResNetV2, and InceptionV3. The model DenseNet201 had 99.64% accuracy. However, all the three studies above admitted having limitations like the need for substantial computation, dependence on huge labeled data sets, restriction in leaf-based identification systems, and inconsistencies with geographic data sets. A comprehensive research carried out in 2024 (Tiwari et al., 2023) brought out other problems like the lack of explanatory models, lack of methodologies, and lack of data set in poor countries.

The implementations had limitations on scalability but regionally feasible. In 2023 (Saranya et al., 2024), ResNet50 (95.79%) and VGG19 (97.6%) were used for classification of 64 plants of Kerala by integrating usage instructions and safety. Using the light-weight architecture of the CNN that can be applied even in mobiles, Pushpa and Rani, (2023) created Ayur-PlantNet for classification of 40 plants from Ayurveda. Regional limitations, special lighting needs, small range of plants (40–64 versus more than 3,000 medicinal species in India), and generalization failure unless re-trained restricted their usefulness. Other approaches such as OTAMNet (2022) (Oppong et al., 2022) using Log-Gabor filter with 98% accuracy rate and low FP (false positives, < 0.1%), suffered under real field conditions (water droplets, dust, damages to leaf).

Plant-CNN-ViT, a combination of Vision Transformer and CNNs (ResNet-50, DenseNet-201, Xception) developed by Lee et al. (2023), leverages the strengths of attention and hierarchical feature extraction through an ensemble learning approach. The proposed model demonstrated perfect accuracies of 100% for the Flavia, Folio, and Swedish Leaf datasets and 99.83% for the MalayaKew dataset, proving high efficiency even when dealing with small-size training datasets (with only 14–45 examples per class). Wagle et al. (2022) designed three efficient CNN-based classifiers (N1, N2, N3), which delivered 99.45%–99.65% accuracies on the PlantVillage dataset using less computational resources than other models. Models N1 and N3 were respectively 92.67% smaller (with a size of 14.8 MB) compared to AlexNet (202 MB), taking 34% shorter time to train while maintaining the same level of accuracy. Nevertheless, both studies admit some limitations in their research despite excellent results, such as dependence on controlled environment, lack of experimental validation with field photos with various backgrounds, and limitation to leaf recognition without medicinal information.

Advances in transformer models for computer vision have led to improved fine-grained medicinal plant recognition owing to self-attention mechanisms. While conventional CNN models depend on local spatial features extracted using convolutions, Vision Transformers (ViT) and other variants can capture distant relationships in different parts of an image, which helps discriminate visually similar plants from one another. As per the findings of Tran Nguyen Nhut et al. (2024), the ViT and BEiT models are better at extracting features as compared to traditional CNN models in medicinal plant recognition applications. In addition, current literature has witnessed a trend towards using hybrid architectures of AI that utilize both the strength of CNN based architectures in terms of localized feature extraction and the strength of transformer based architecture with regards to their ability to understand the global context of an image.

Firdous et al. (2025) designed a hybrid AI framework incorporating a pre-trained Vision Transformer (ViT-B16) alongside a CatBoost classifier for recognition of Indian medicinal plants. Through the use of multi-headed self-attention algorithms, the model was successful in capturing long range spatial dependency features, making it robust under field conditions due to its ability to overcome limitations of classical CNNs. With their hybrid algorithm, they were successful in achieving 93.3% accuracy in classification. Plant-CNN-ViT was also designed by Lee et al. (2023), which incorporated both CNN and transformer attention models to achieve a balance between localized texture features and global context. Recently, transformer models such as Swin Transformer have gained popularity due to their hierarchical shifted window attention architecture which allows for reduced computational complexity with retention of multi-scale contextual information (Tran Nguyen Nhut et al., 2024), (Lee et al., 2023). Current research has introduced MediFlora-Net (Uma et al., 2026), which is a highly accurate multimodal deep learning-based model that incorporates both RGB and hyperspectral images for the identification of medicinal plants. This model has been developed using GAN based data augmentation, quantum-inspired feature extraction, enhanced CNNs, and Vision Transformers to achieve 99.52% accuracy while ensuring negligible misclassification errors. Through hybrid ensembling and robust feature representation, the proposed model has shown robustness with respect to different imaging techniques. Nevertheless, its practical applicability is hampered due to its computational complexity and dependence on expensive hardware infrastructure.

However, the main distinction lies in the basic disparity between an identification system and a therapeutic information system that gives only the name of the plant species without phytochemical content or uses.

## 3 System design and Methodology

The system proposed in this paper is a comprehensive multiple-user platform to detect medicinal plants and extract relevant information. This system is formulated in such a way that it forms a closed-loop intelligent ecosystem, which integrates cutting-edge deep learning for instantaneous plant identification with the robustness of a human-in-the-loop (HITL) curation process.

### 3.1 Data materials and methods

The species selection was performed by considering a minimum requirement of 1,000 images per species based on the PlantNet-300K dataset, while species with less than 1,000 images per class did not have enough images for performing deep learning, which had varying image counts between 1 and several hundred images. The inclusion criteria further required each classified species to have validated profiles in the IMPPAT and OSADHI databases. The PlantNet-300K dataset (Garcin et al., 2021) was used for model development.

The FloraMediX approach relies on a wide range of resources that contribute to its well-established knowledge base, showing how varied data collection can be and how each type of resource can be useful for the system. Different sources, including the scientific database of medicinal plants known as IMPPAT (Database) and OSADHI (CSIR–North East Institute of Science and Technology, 2026), provide scientific, common, and local information about plants. Images from PlantNet datasets (Badoa, 2023), including 75 plant species in total, are used for training and validation of the model and are part of the application. Another important source that is included in the app is the PlantNet API (PlantNet Consortium, 2026), which allows to classify new images instantly; the model itself is also used for classification tasks. Currently, the PlantNet API is the main inference engine with the locally trained Swin-B model running simultaneously to act as a comparative baseline for performance evaluation. Inferences generated by both systems for a single image are recorded separately. This approach to inference generation has a long-term strategy behind it–the performance difference between the locally trained model and the PlantNet API is utilized to detect wrongly classified or ambiguous images, which go through a human-in-the-loop process and become part of the re-training dataset. As a result of using this process iteratively, the locally trained Swin-B model can gradually improve its performance to catch up with that of the PlantNet API, making itself a replacement candidate to act as the main inference engine. Finally, the Geographical information is provided by Humanitarian Data Exchange (HDX) (The Humanitarian Data Exchange, 2025).

**FIGURE 1:**
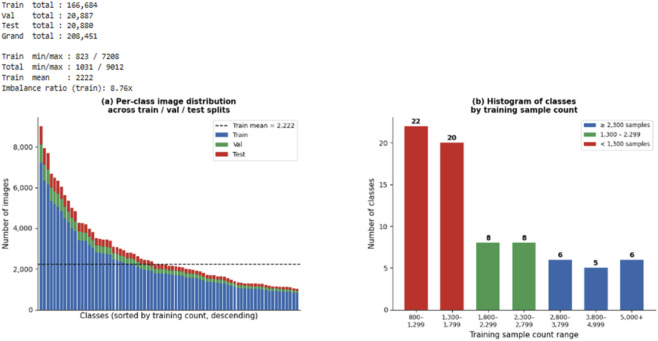
Per-class training sample distribution across 75 medicinal plant species. **(a)** Bar chart sorted in descending order; dashed line indicates the mean (2,222). **(b)** Histogram of class frequency by sample count bin.

The dataset used in the system consists of 208,451 pictures which belong to 75 classes. The data sources used in FloraMediX are summarized in Table 1. With regard to PlantNet-300K splits predefined, 166,684 images (79.9%), 20,887 images (10.0%), and 20,880 images (10.0%) are allocated to the training, validation, and testing sets respectively in 75 classes, ensuring that there is no overlapping between them. It is found that an obvious imbalance exists among all classes in terms of training set through analysis, where sample sizes are varied from 823 to 7,208 images for each class (average: 2,222; class imbalance ratio: 8.76:1). As illustrated by Figure 1, most of the classes belong to the group between 800 and 2,200 images per class while there are only six classes whose sample numbers are greater than 5,000.

**TABLE 1:** Data sources table.

Resources	Type of data	Purpose
IMPPAT (Database, 2025)	Textual/ Botanical Data	Provides the core botanical and medicinal information for the knowledge base, including scientific names, chemical constituents, and therapeutic uses.
Osadhi (CSIR–North East Institute of Science and Technology, 2026)	Geospatial/ Location Data	Supplies the geographical location data used for mapping the distribution of different plant species.
Humanitarian Data Exchange (Hdx) (The Humanitarian Data Exchange, 2025)	Geospatial Map Data (JSON)	Provides the base map file (in JSON format) required to build the interactive location and distribution maps within the application.
PlantNet-300KDataset (Badoa, 2023)	Image Data	Image dataset for training and validating the image classification model for 75 classes.

**FIGURE 2:**
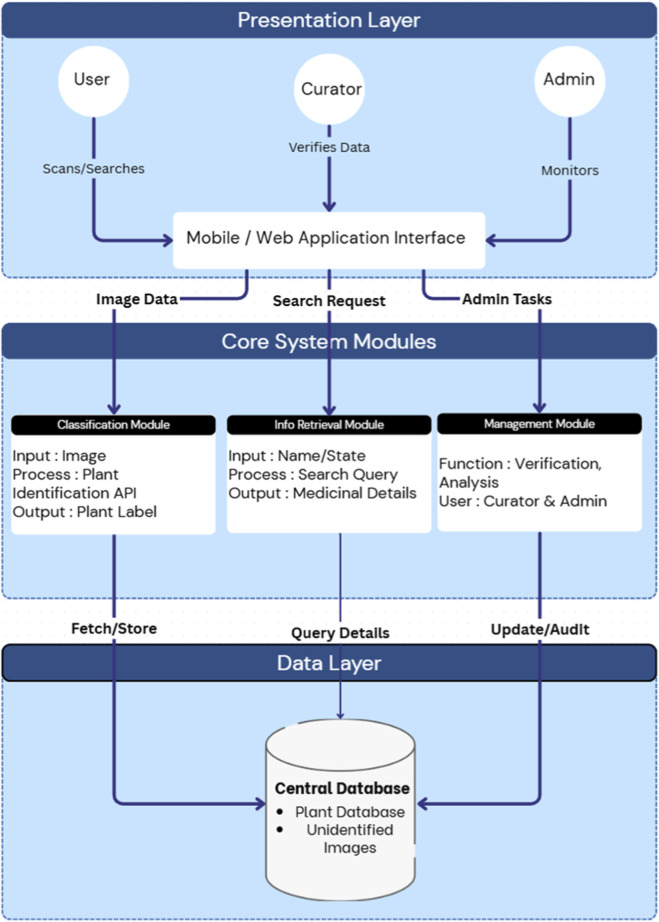
System architecture of FloraMediX system.

As concerns the spatial resolution of the images, it ranges between 437 × 437 pixels and 600 × 600 pixels. Each picture in the dataset is stored in the form of.jpg files and represented as full RGB images. The size of the files varies approximately between 50 KB and 190 KB. It should be stressed that the dataset represents a good and reliable basis for developing deep learning models for recognizing medicinal plants. The integrity of the experimental results was guaranteed by strictly distinguishing the training and validation datasets. The division of the dataset was done according to the pre-defined splits in the PlantNet-300K dataset. It was ensured that no image belonged to both the training and validation datasets to prevent any form of data leakage that may enable the learning algorithm to indirectly utilize the information contained in the validation set during the training process. Furthermore, the balance of class representation in both datasets was considered without duplicating the images. The overall architecture of the FloraMediX system is illustrated in Figure 2.

Because of the crowd-sourced nature of PlantNet-300K, there may be some annotations that have noisy or even wrong labels. In order to alleviate the problem, label smoothing was used with coefficient equal to 0.1 on the cross-entropy loss such that the network is able to spread some probability to other than target classes. Uncertainties in the output predictions when deployed are additionally addressed via human-in-the-loop curation process explained in Section III-C.

### 3.2 System architecture

The proposed system follows a modular approach that seeks to integrate plant recognition capabilities through automation while verifying its curation process through human validation. It begins with the Input Module, where plant images can be uploaded or scanned and held in temporary storage before being processed. This data then goes to the Core Logic Module, which interfaces with the external PlantNet API for species prediction. Based on this interaction, the system will return predictions based on the highest likelihood, five other top picks, and their respective levels of certainty, or “Unknown” if the confidence level is very low.

The Decision Checkpoint Module evaluates the results generated by the API. High-confidence predictions automatically trigger the process of acquiring information from the system’s knowledge base. This is followed by the Results Mapping Module, wherein relevant information regarding botany, pharmacology, and geography is extracted before showing it to the user. In case the system cannot accurately identify the plant, then the plant’s picture will be redirected to the System Improvement and Verification Loop. During this process, unidentifiable images will be flagged and passed through the Curator Module for analysis where specialists will provide classifications. The proposals from the curator will then be validated by the Senior Curator who releases them to be incorporated in the Knowledge Base used in production and a Retraining Dataset for updating the internal model through retraining.

A User Interaction Module is provided by the system that allows users to search for information on plant species using images and text as well as filtering by states. This module is mainly built on the Classification Module that utilizes deep learning techniques for analyzing images automatically as well as the Information Retrieval Module providing structured data regarding plants for classification.

### 3.3 Methodology

**FIGURE 3:**
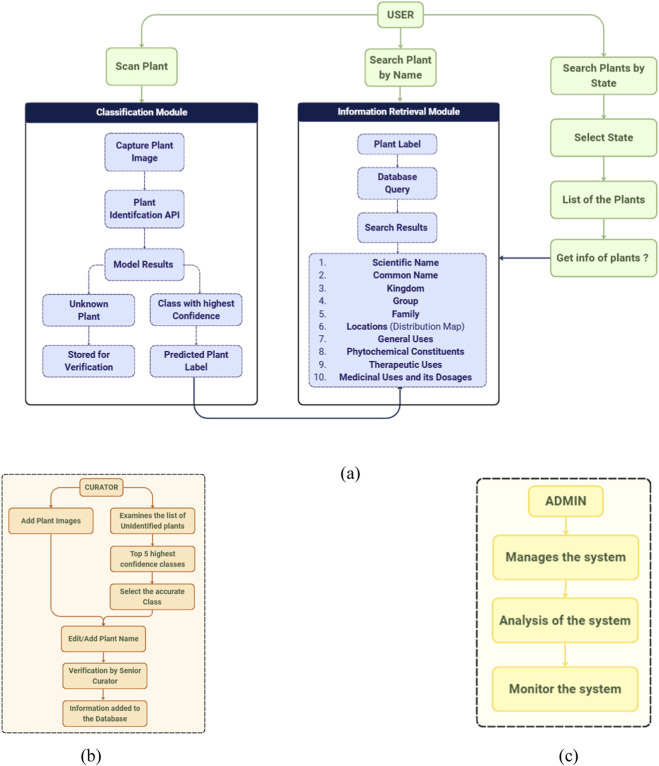
Modular Architecture of the FloraMediX System: **(a)** User Module illustrating plant classification and information retrieval workflows; **(b)** Curator Module depicting expert validation and dataset augmentation processes; **(c)** Admin Module representing system monitoring and management functionalities.

The idea behind the modular architecture of the FloraMediX system is to achieve scalability, reliability, as well as collaborative work between humans and artificial intelligence. The User Module is able to perform plant recognition by capturing the images using a camera on smartphones or computers, then applying both model-based classification as well as validation using API and getting detailed data. The Curator Module incorporates a human-in-the-loop approach, whereby specialists have the ability to review any unclear results, validate them and make the database better in order to improve future models. The Admin Module deals with all system-wide operations such as monitoring performance, managing workflows and other activities related to overall system maintenance. The system’s ability to learn iteratively, as well as increase its accuracy through this process, will contribute to increasing its resilience due to the modular architecture with multiple layers. The workflow described in Figure 3a begins from the Input Module, where users are supposed to upload their images from the smartphone or computer into the Input Module. Then, the image is kept in a particular memory buffer for further processing. Finally, the stored image is passed to the Core Logic Module, which performs real-time plant recognition.

The PlantNet API Call acts as the primary external inference engine in the system. Once the image has been processed, a prediction from the API call entails the most probable plant species along with its probability score, a ranked listing of the top three probable plants, as well as the “Unknown” tag when all probability scores fall below a certain threshold. Following the assessment of this API result, the Decision Checkpoint Module ensures that only those predictions with sufficient probabilities proceed to the next stage of the process. As soon as the API makes the right prediction about the plant under consideration and presents an adequate probability score, the predicted plant species is accepted, and the system proceeds to the information-gathering stage. However, when there is uncertainty about or even failure in the prediction stage, the image undergoes a specific verification process.

In order to obtain correct predictions, it is important to use the validated name of the plant since it helps to become an important component of the Knowledge Base access, which comprises structured data about the plant, including its medicinal uses, geographical distribution, and chemical composition. The information related to the selected plant is collected by the Search and Details Extraction Module, after which the data is organized by the Results Mapping Module.

In the event that the system cannot identify an image correctly, the flow goes into the System Improvement and Verification Loop, a human in the loop approach that is used to improve the performance of the machine learning model over time. Here, unidentified images and other inputs provided by users are flagged for further specialist review. The Curator Module (Figure 3b) is used for identification and validation of plant samples through a hierarchical approach involving domain experts in two levels. The experts that participate in this phase include curators who are well trained on botany, plant taxonomy or biological sciences generally, and are capable of analyzing the traits of plants using images. In the first level, the Junior Curators (annotators and other individuals with basic training in botany) will be responsible for analyzing the plant image data and providing a tentative classification or marking it as unidentified. After analyzing this information, the revised data will then be passed into the next stage where the Senior Curators (botanists or taxonomists) will confirm the identification.

The Admin Module, shown in Figure 3c, serves as the primary management dashboard for overseeing the entire system and users. Rather than focusing on the validation of each plant entry, the role of the Admin revolves around more general management responsibilities, such as assigning roles (assigning Junior Curators and Senior Curators, for example,) and monitoring the analytics of the platform. The senior Curators are responsible for verifying plant data that leads to the creation of the knowledge base, and the Admin Module provides the dashboards for this purpose.

The entire system’s user interface is controlled through the User Interaction Module, which facilitates numerous ways of accessing information, such as identifying plants through images, searching plants by name, and finding information based on state.

The design of the model involves the incorporation of a fusion strategy for plant recognition based on the integration of a deep learning classifier and an external plant recognition API. In regard to the deep learning model, the model will leverage the power of the Swin Transformer Base version (Swin-B) architecture that is pre-trained on the ImageNet-1K benchmark set at 384 × 384 input resolution. The architecture splits input image resolutions into 4 × 4 patches to create an embedding space of 128 dimensions. In turn, features pass through four layers with a depth of (National Medicinal Plants Board, 2026b; Oppong et al., 2022; National Medicinal Plants Board, 2026b) while applying multilayer perceptron (MLP) expansion ratios of [4, 8, 16, 32]. The overall number of parameters within the model comes to around 87.9 million. Specifically, the pre-trained classification layer was swapped with a fully connected linear output layer of 75 neurons, and the whole network was trained end-to-end using the Lion optimization algorithm with a cosine annealing schedule. In terms of training, the training process will be conducted offline using samples from the PlantNet-300K dataset after preprocessing.

In the deployment stage, the operation of the system takes place through a two-stage inference pipeline. When a plant image is uploaded by the user, both requests are simultaneously sent to the PlantNet API and the Swin transformer that has been trained and is being hosted locally. The Swin transformer makes use of the neural network to predict the type of plant and the confidence level of such prediction, while PlantNet provides an additional classification with the help of its own database. This combination improves the performance and robustness of plant recognition by the system. After training, the Swin transformer serves as a backend inference model accessible through an API.

The whole system is supported by the robustness of the Curation and Administration Subsystem, which provides data consistency, improves models, and ensures scalability in operations. The Admin oversees the performance of the system and makes final judgments, whereas Curators make sure that there is scientific accuracy in the process by verifying the predictions and enriching the database with additional information. All these components together form a continually developing plant identification system.

## 4 Results

In this section, we examine the performance results obtained after training and testing of the FloraMediX model. The performance measures include parameters such as training time, loss values, and validation accuracy among others. In addition, we evaluate the accuracy graphs and the loss graphs to establish learning convergence.

This process was conducted using a HPC cluster with GPU acceleration, where the job scheduling was done by using the Portable Batch System (PBS). The training of this model was done by using an NVIDIA A40 GPU (with 48 GB of VRAM), along with 10 vCPUs and 64 GB RAM. This entire process took about 48 h to complete.

In Table 2, there is a comparison between the four architectures that have been proposed according to the parameters of the training time, the convergence, the loss value, and the validation accuracy. It is evident that among all the architectures, the highest validation accuracy was acquired by the Swin Transformer, which is 93.81%. This shows that the Swin Transformer can represent the features better compared to the other three architectures, despite having a lengthy training time per epoch. In CoAtNet, the effectiveness was found to be very high (92.22%). The training time was comparatively lesser than that of the Swin Transformer. Thus, it indicates the effective fusion of convolution and attention-based models. Similarly, the accuracy obtained from ConvNeXt is very high; however, it required many more epochs to converge than the other models. On the contrary, ResNet-CBAM had the shortest training time; however, the validation accuracy was the least among the four models, which is 90.57%.

**TABLE 2:** Comparison of image classification models.

Model name	Epochs	Training time/ Epoch (in minutes)	Train loss	Validation loss	Validation accuracy
Swin Transformer	22	120	1.2542	0.9154	0.9381
CoAtNet	13	60	0.2142	0.3274	0.9222
ConvNeXt	38	45	2.1226	1.0474	0.9135
Resnet-CBAM	20	60	0.2151	0.3207	0.9057

Swin Transformer follows a hierarchical architecture where localized self-attention is done in each window individually, resulting in much lower complexity but maintaining spatial information. Through the shift mechanism, the windows are able to communicate better, and as such, capture relationships in the local and overall scope. This is advantageous in that different levels of abstraction are possible from the model and therefore is ideal when dealing with visual classification problems. The outstanding success seen in this particular case is mainly due to its ability to model and differentiate fine differences in the plant properties.

**FIGURE 4:**
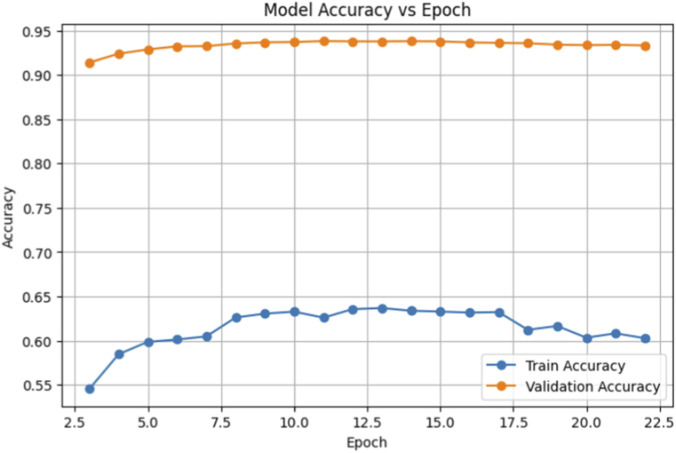
Accuracy vs Epochs Comparison.

**FIGURE 5:**
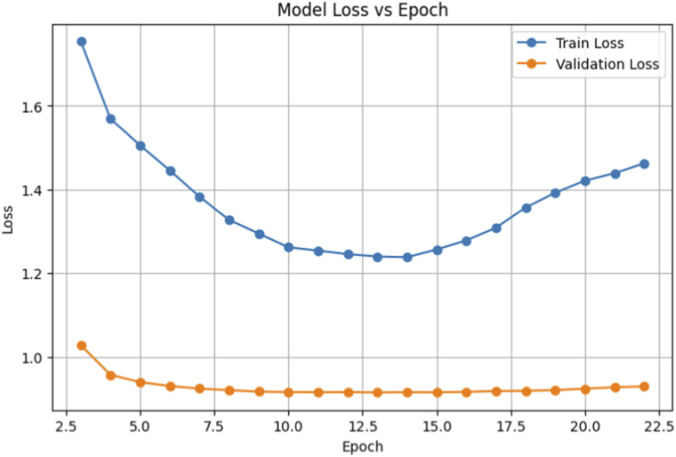
Loss vs Epochs Comparison.

Figure 4 demonstrates the fluctuation of the training and validation accuracies as a function of the number of epochs, while Figure 5 shows the loss curves. It is important to highlight that, during training, the validation accuracy was always greater than the training accuracy, going from 0.91 to 0.94; meanwhile, the training accuracy went from 0.54 to 0.64. The reason behind such results might be mainly associated with the use of powerful regularization methods and data augmentation when training the model. The technique of data augmentation makes the training dataset harder and more diverse since transformations, including rotation, flip, rescaling, and colors alterations, are performed before feeding the samples into the neural network. This means that, due to increased difficulty in learning, the training accuracy is lower than the validation accuracy because the former uses more challenging samples. Moreover, the implementation of powerful regularization techniques, like dropout, during training makes it harder for the model to learn from data.

Thus, the increased validation accuracy does not signify any problem of overfitting, but it shows that the model’s performance on unseen data has been enhanced due to training under regularization. Moreover, the loss functions have further reinforced this conclusion. As seen, there is a steep decrease in the training loss function to a minimum in the 10th to 12th epochs, signifying effective convergence due to parameter optimization. Subsequent marginal increases may be due to augmentation and regularization. Similarly, the validation loss function shows a decline from 1.02 to 0.92 without overfitting.

Hence, the best model has been achieved during the 11th epoch when the validation accuracy becomes maximal with a value of 0.9381.

**FIGURE 6:**
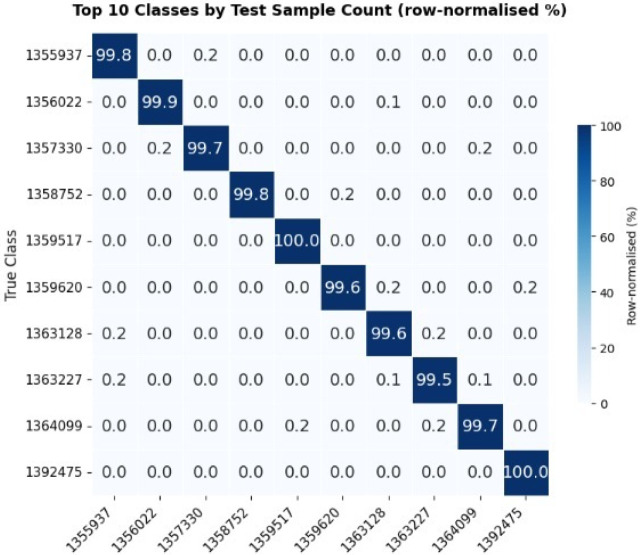
Confusion matrix of Top 10 classes by Test Sample Count (row-normalised %).

For a thorough performance analysis of the proposed model, classification performance measures were calculated on the independent test set of 20,880 images. As shown in Table 3, the Swin-B architecture showed an impressive top-1 accuracy of 93.70% and a top-5 accuracy of 99.68%, indicating robust discrimination among all 75 categories. The macro-averaged values of precision, recall, and F1 score for the model were 91.10%, 91.93%, and 91.38%, respectively. Macro-averaged specificity also turned out to be very high at 99.91%. The value of the macro-averaged AUC of the model in the one-vs-rest setting is 0.9987, signifying the accurate segregation of all species into different categories. Figure 6 shows the confusion matrix of top 10 classes by test sample count; diagonal dominance means consistent correct classifications, while off-diagonal errors are mostly between similar species.

**TABLE 3:** Evaluation metrics of the FloraMediX Swin-B model on the held-out test set.

Metric	Values
Top-1 Accuracy	93.70%
Top-5 Accuracy	99.68%
Macro Precision	91.10%
Macro Recall	91.93%
Macro F1-score	91.38%
Macro Specificity	99.91%
Macro AUC (OvR)	0.9987

**FIGURE 7:**
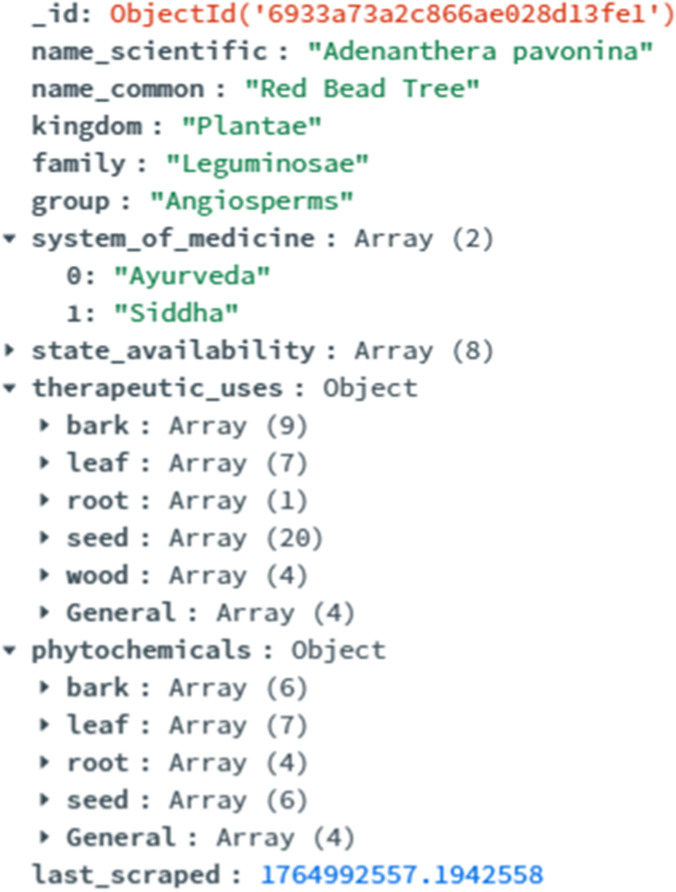
Schema representation of the plant collection in the MongoDB database.

As shown in Figure 7, the schema of the plant collection which is stored in the MongoDB database has been designed for the purpose of ensuring efficient data management of the large volume of information regarding medicinal plants. Every document represents an individual record about a certain plant occurrence and carries relevant taxonomic information, such as the scientific name, common name, kingdom, family, and plant group. The schema employs arrays to allow for a variety of possible values when referring to features like the medical system and distribution.

Moreover, the database consists of a complete dataset with regard to the purpose of treatment and the components of the plants according to the phytochemical compounds found in them. The information regarding the components of the plants is categorized according to the parts of the plant, including bark, leaves, roots, seeds, and wood. Moreover, there is other metadata in the database, including the date of the last scrape.

**FIGURE 8:**
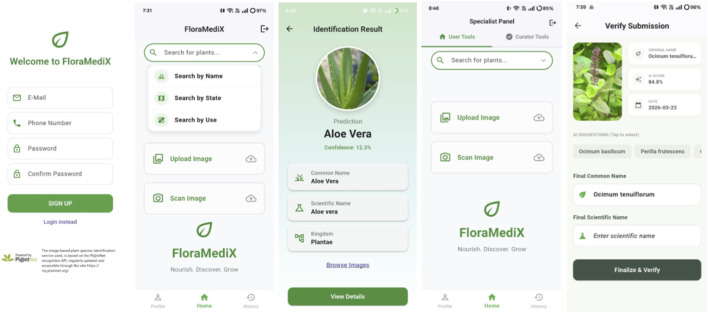
User interface Screens of the FloraMediX application demonstrating Core functionalities.

Figure 8 demonstrates the GUI of the FloraMediX application with a focus on some of the interactions made by users along with features provided by the system. Some of the notable features include user login options, searching for plants based on their names, conditions, or usage, as well as recognizing plants based on pictures. In particular, the interface used for identifying plants emphasizes the implementation of the deep learning model through its results with confidence scores and botany information included.

Additionally, the curator validation section provides human involvement into the process of validating predictions of the model, which increases its accuracy. As evident from the image below, the process of uploading images and scanning functions along with submission validation processes are examples of user interactions with professionals who can be considered curators of this platform.

The input images show noticeable variations in angle, lighting, background complexity, scale, and occlusion. However, in all cases, the model demonstrates an ability to determine the correct plant species with high precision, thereby showing great generalization abilities. This suggests that the use of data augmentation methods along with the hierarchical feature extraction process in the Swin Transformer allows identifying visual features of plants, namely, their leaves, edges, and textures. Thus, the system operates effectively even under nonideal conditions of the real world.

## 5 Discussion

The existing plant recognition systems such as Pl@ntNet or other generic visual classifiers are mainly used as prediction systems that classify images according to the species’ names of plants. Although this approach is useful for basic identification purposes, it fails in providing structured knowledge about the plant’s botanical or medicinal properties and does not have any mechanisms that could be used for correcting errors. Moreover, this type of approach usually works as a static algorithm and does not improve or change according to any new information. In contrast, the system presented in this paper is built as a combination of a deep learning classifier with the Swin Transformer, as well as with a knowledge database containing medicinal properties of different plants. To ensure data integrity, the system includes an additional human-in-the-loop step when predictions are manually validated by Junior and Senior Curators. Besides, to further improve predictions’ robustness and confidence, hybrid prediction that uses results obtained both by training model and Pl@ntNet API is considered.

Our model shows that the method works really well for identifying plant species. The Swin-B model got tested on almost 21,000 images, and here are the scores: Top-1 accuracy was 93.70%, Top-5 was 99.68%, macro F1-score was 91.38%, precision was 91.10%, recall was 91.93%, specificity was 99.91%, and AUC was 0.9987. Compared to other models like ResNet-CBAM and ConvNeXt, Swin-B did way better. Even CoAtNet couldn't match its success. This proves that Swin-B’s shifted-window attention mechanism does an excellent job for fine plant detail recognition compared to regular CNN methods.

Apart from that, the database contains information regarding the purpose of treatment and plant content based on the phytochemical compound. The information regarding the plants is categorized according to various parts of the plant, such as bark, leaves, roots, seeds, and wood. Metadata is contained in the database, which includes the latest date when the particular dataset was scraped.

## 6 Conclusion and future scope

The success of the FloraMediX system proves that the integration of artificial intelligence with databases of real medicines guarantees precise and comprehensive identification and analysis of medicinal plants. Our model got tested on 20,880 images and aced the test with 93.70% for Top-1 accuracy, 99.68% for Top-5 accuracy, plus a macro F1-score of 91.38% and an AUC of 0.998. The outcomes of the sensitivity analysis support the reliability of the system with regard to variation in the image quality, while the administration and information modules ensure the provision of accurate and updated information to the users. FloraMediX encourages further research into digital botany and inspires more people to contribute to research into the heritage of medicinal plants of India. Future improvements of FloraMediX would be oriented toward extension of the range of applications. The main improvement of the system would consist in introducing a smart chatbot, which would guarantee personalized recommendation of plant materials. In other words, with the use of the system, users will have the opportunity to input a human disease, after which the system will output a set of recommended medicinal plants with specified doses. Moreover, there will be an emphasis on making the models even more accurate and optimized so that species can be identified with greater precision. There will also be enhancements in scalability so that all users can be served simultaneously with no latency involved whatsoever. Future work will use explainable AI, like Grad-CAM and Swin Transformer, to make models clearer and more trustworthy for possible uses in medicine. Such attributes will enable FloraMediX to grow from its present form into one that is much more intelligent, offering a wider range of services and educating people about medicinal plants.

## Data Availability

The original contributions presented in the study are included in the article/supplementary material, further inquiries can be directed to the corresponding author.
